# Situating emotional experience

**DOI:** 10.3389/fnhum.2013.00764

**Published:** 2013-11-26

**Authors:** Christine D. Wilson-Mendenhall, Lisa Feldman Barrett, Lawrence W. Barsalou

**Affiliations:** ^1^Department of Psychology, Northeastern UniversityBoston, MA, USA; ^2^Department of Psychology, Emory UniversityAtlanta, GA, USA

**Keywords:** emotion, situated cognition, affective neuroscience, affect, cognitive neuroscience

## Abstract

Psychological construction approaches to emotion suggest that emotional experience is situated and dynamic. Fear, for example, is typically studied in a physical danger context (e.g., threatening snake), but in the real world, it often occurs in social contexts, especially those involving social evaluation (e.g., public speaking). Understanding *situated* emotional experience is critical because adaptive responding is guided by situational context (e.g., inferring the intention of another in a social evaluation situation vs. monitoring the environment in a physical danger situation). In an fMRI study, we assessed situated emotional experience using a newly developed paradigm in which participants vividly imagine different scenarios from a first-person perspective, in this case scenarios involving either social evaluation or physical danger. We hypothesized that distributed neural patterns would underlie immersion in social evaluation and physical danger situations, with shared activity patterns across both situations in multiple sensory modalities and in circuitry involved in integrating salient sensory information, and with unique activity patterns for each situation type in coordinated large-scale networks that reflect situated responding. More specifically, we predicted that networks underlying the social inference and mentalizing involved in responding to a social threat (in regions that make up the “default mode” network) would be reliably more active during social evaluation situations. In contrast, networks underlying the visuospatial attention and action planning involved in responding to a physical threat would be reliably more active during physical danger situations. The results supported these hypotheses. In line with emerging psychological construction approaches, the findings suggest that coordinated brain networks offer a systematic way to interpret the distributed patterns that underlie the diverse situational contexts characterizing emotional life.

## INTRODUCTION

Darwin’s *The Expression of the Emotions in Man and Animals* is often used to motivate emotion research that focuses on identifying the biological signatures for five or so emotion categories (Ekman, 2009; Hess and Thibault, 2009). Interestingly, though, the evolution paradigm shift initiated by Darwin and other scientists heavily emphasized *variability*: species are biopopulations in which individuals within a population are unique and in which individual variation within a species is meaningfully tied to variation in the environment (and they are *not* physical types defined by essential features; Barrett, 2013). In other words, an individual organism is best understood by the situational context in which it operates. It is not a great leap, then, to hypothesize that “situatedness” is also a basic principle by which the human mind operates, during emotions and during many other mental phenomena (Barrett, 2013).

Situated approaches to the mind typically view the brain as a coordinated system designed to use information captured during prior situations (and stored in memory) to flexibly interpret and infer what is happening in the current situation – dynamically shaping moment-to-moment responding in the form of perceiving, coordinating action, regulating the body, and organizing thoughts (Glenberg, 1997; Barsalou, 2003, 2009; Aydede and Robbins, 2009; Mesquita et al., 2010; Barrett, 2013). “Cognitive” research domains (e.g., episodic and semantic memory, visual object recognition, language comprehension) are increasingly adopting a situated view of the mind (for empirical reviews, see Zwaan and Radvansky, 1998; Barsalou, 2003; Bar, 2004; Yeh and Barsalou, 2006; Mesquita et al., 2010). In contrast, emotion research largely remains entrenched in a “stimulus-response” reflexive approach to brain function, which typically views the brain as reacting to the demands of the environment, often in a simple, stereotyped way (cf. Raichle, 2010). Traditional “basic” emotion views often assume that an event (i.e., a stimulus) triggers one of several stereotyped responses in the brain and body that can be classified as either fear, disgust, anger, sadness, happiness, etc. (for a review of basic emotion models, see Tracy and Randles, 2011). Decades of research have revealed substantial variability in the neural, physiological, and behavioral patterns associated with these emotion categories (cf. Barrett, 2006; Lindquist et al., 2012). Whereas basic emotion approaches now focus on trying to identify primitive “core” (and often narrowly defined) instances of these emotions, alternative theoretical approaches to emotion, such as psychological construction, propose taking a situated approach to explaining the variability that exists in the experiences people refer to using words like fear, disgust, anger, sadness, happiness (and using many other emotion terms; Barrett, 2009b, 2013).

In the psychological construction view that we have developed, emotions are not fundamentally different from other kinds of brain states (Barrett, 2009a, 2012; Wilson-Mendenhall et al., 2011). During emotional experiences and during other kinds of experiences, the brain is using prior experience to dynamically interpret ongoing neural activity, which guides an individual’s responding in the situation. We refer to this process, which often occurs without awareness (i.e., it is a fundamental process for making sense of one’s relation to the world at any given moment), as *situated conceptualization*. The term *situated* takes on a broad meaning in our view, referring to the distributed neural activity across the modal systems of the brain involved in constructing situations, not just to perception of the external environment or to what might be considered the background. More specifically, situated neural activity reflects the dynamic actions that individuals engage in, and the events, internal bodily sensations, and mentalizing that they experience, as well as the perceptions of the external environmental setting and the physical entities and individuals it contains (Wilson-Mendenhall et al., 2011).

Emotions, like other classes of mental experiences, operate in this situation-specific way because rich, cross-modal knowledge is critical for interpreting, inferring, and responding when similar situations occur in the future. On this view, situational knowledge develops for emotion categories like fear, anger, etc., as it does for other abstract categories of experiences (e.g., situations that involve the abstract categories gossip, modesty, or ambition). Experiences categorized as fear, for example, can occur when delivering a speech to a respected audience or when losing control while driving a car. A situated, psychological construction perspective suggests that it is more adaptive to respond differently in these situations, guided by knowledge of the situation, than to respond in a stereotyped way. Whereas responding in the social speech situation involves inferring what audience members are thinking, responding in the physical car situation involves rapid action and attention to the environment. Stereotyped responding in the form of preparing the body to flee or fight does not address the immediate threat present in either of these situations. A psychological construction approach highlights the importance of studying the situations commonly categorized as emotions like fear or anger, not because these situations merely describe emotions, but because emotions would not exist without them.

**FIGURE 1:**
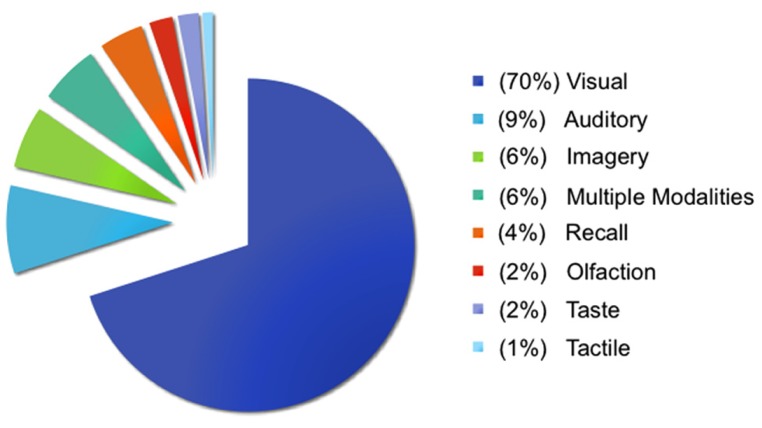
**Methods used to study emotion and affect.** Visual methods typically involved viewing faces, pictures, films, words, sentences, and/or bodies. Auditory methods typically involved listening to voices, sounds, music, words, and/or sentences. Imagery methods typically involved generating imagery using personal memories, sentences, faces, and/or pictures (and are described further in the main text). Recall methods typically involved recalling personal events, words, films, or pictures. Tactile methods involved touch or thermal stimulation, olfaction methods involved smelling odors, and taste methods involved tasting food. Multiple modalities refers to studies that involved two or more of the aforementioned methods in the same study, with visual and auditory methods being the most frequent combination.

A significant challenge in taking a situated approach to studying emotional experience is maintaining a balance between the rich, multimodal nature of situated experiences and experimental control. Immersion in emotional situations through vividly imagined imagery is recognized as a powerful emotion induction method for evoking physiological responses (Lang et al., 1980; Lench et al., 2011). Imagery paradigms were initially developed to study situations thought to be central to various forms of psychopathology (Lang, 1979; Pitman et al., 1987), and remain a focus in clinical psychology (for a review, see Holmes and Mathews, 2010). In contrast, a small proportion of neuroimaging studies investigating emotion in typical populations use these methods. **Figure 1** illustrates the methods used across 397 studies in a database constructed for neuroimaging meta-analyses of affect and emotion (Kober et al., 2008; Lindquist et al., 2012)^1^. Visual methods dominate (70% of studies), with the majority of these studies using faces (42% of visual methods) and pictures (36% of visual methods) like the International Affective Picture System (IAPS; Lang et al., 2008). In contrast, only 6% of studies have used imagery methods^2^. Imagery methods appear to be used more frequently when studying complex socio-emotional experiences that would be difficult to induce with an unfamiliar face or picture and that are often clinically oriented, including angry rumination (Denson et al., 2009), personal anxiety (Bystritsky et al., 2001), competition and aggression (Rauch et al., 1999; Pietrini et al., 2000), social rejection and insult (Kim et al., 2008; Kross et al., 2011), romantic love (Aron et al., 2005), moral disgust (Moll et al., 2005; Schaich Borg et al., 2008), and empathy (Perry et al., 2012).

Imagery-based neuroimaging studies of emotional experience typically take one of two approaches. The most frequent approach is to draw on the personal experiences of the participant, cueing specific, vivid memories in the scanner. Often participants’ personal narratives are scripted and vividly imagined (guided by the experimenter) outside the scanner, and then a version of this script is used to induce these memory-based emotional experiences during neuroimaging (e.g., Bystritsky et al., 2001; Marci et al., 2007; Gillihan et al., 2010). Less often, a specific visual stimulus is potent enough to easily evoke personal, emotional imagery in the scanner (e.g., face of a romantic partner; Aron et al., 2005; Kross et al., 2011). The second approach is to present standard prompts (e.g., a sentence) that participants use to generate imagery underlying emotional experiences (e.g., Colibazzi et al., 2010; Costa et al., 2010). A key strength of the first approach is that emotional experiences are tightly tied to situated, real-life memories, whereas a key strength of the second approach is the experimental control afforded by presenting the same prompts to all participants. In both cases, though, the situational context of the emotional experiences is typically lost, either because the situational details are specific to the individual (and thus lost in group-level analyses) or because standard prompts are not designed to cultivate and/or systematically manipulate the situational context of the emotional experience.

Building on the strengths of existing imagery-based approaches, we developed a neuroimaging procedure that would allow us to examine participants’ immersion in rich, situated emotional experiences while maximizing experimental control and rigor. In our paradigm, participants first received training outside the scanner on how to immerse themselves in richly detailed, full paragraph-long versions of emotional scenarios from a first-person perspective. The scenarios reflected two ecologically important situation types in which emotional experiences are often grounded: social evaluation and physical danger. Every scenario was constructed using written templates to induce a social evaluation emotional experience or a physical danger emotional experience (see **Table 1** for examples). Participants listened to audio recordings of the scenarios, which facilitated immersion by allowing participants to close their eyes. In the scanner, participants were prompted with shorter, core (audio) versions of the scenarios in the scanner, so that a statistically powerful neuroimaging design could be implemented.

**Table 1:** Examples of physical danger and social evaluation scenarios used in the experiment.

Examples of physical danger situations
**Full version**
(P1) You are driving home after staying out drinking all night. (S1) The long stretch of road in front of you seems to go on forever. (P2A) You close your eyes for a moment. (P2C) The car begins to skid. (S2) You jerk awake. (S3) You feel the steering wheel slip in your hands.
**Core version**
(P1) You are driving home after staying out drinking all night. (P2) You close your eyes for a moment, and the car begins to skid.
**Full version**
(P1) You are jogging along an isolated lake at dusk. (S1) Thick dark woods surround you as you move along the main well-marked trail. (P2A) On a whim, you veer onto an overgrown unmarked trail. (P2C) You become lost in the dark. (S2) The trees close in around you, and you cannot see the sky. (S3) You feel your pace quicken as you try to run out of the darkness.
**Core version**
(P1) You are jogging along an isolated lake at dusk. (P2) On a whim, you veer onto an overgrown unmarked trail, and become lost in the dark.
**Examples of social evaluation situations**
**Full version**
(P1) You are at a dinner party with friends. (S1) A debate about a contentious issue arises that gets everyone at the table talking. (P2A) You alone bravely defend the unpopular view. (P2C) Your comments are met with sudden uncomfortable silence. (S2) Your friends are looking down at their plates, avoiding eye contact with you. (S3) You feel your chest tighten.
**Core version**
(P1) You are at a dinner party with friends. (P2) You alone bravely defend the unpopular view, and your comments are met with sudden uncomfortable silence.
**Full version**
(P1) You are having drinks at a trendy bar. (S1) The bartender tosses ice cubes into glasses, making a loud clinking sound. (P2A) An attractive stranger strolls by, looks you up and down. (P2C) The stranger walks away smirking. (S2) People around you begin saying that you never meet the right people in bars. (S3) Your cheeks are burning.
**Core version**
(P1) You are having drinks at a trendy bar. (P2) An attractive stranger strolls by, looks you up and down, and walks away smirking.

We hypothesized that immersion across both social evaluation and physical danger situations would be characterized by distributed neural patterns across multiple sensory modalities and across regions involved in detecting and integrating salient sensory information. Much previous research has demonstrated neural overlap between sensorimotor perception/action and sensorimotor imagery (for a review, see Kosslyn et al., 2001). If our scenario immersion method induces richly situated emotional experiences, then the vivid mental imagery generated should be grounded in brain regions underlying sensory perception and action. Perhaps surprisingly, studies using imagery paradigms to investigate emotional experiences do not typically examine sensorimotor activity, because the goal is often to isolate a category of experience (e.g., anger, disgust) or other “emotion” components. In contrast, our approach is designed to examine the distributed neural patterns that underlie emotional experiences.

Our second, primary hypothesis was motivated by a situated approach to studying the varieties of emotional experience. We hypothesized that unique activity patterns for each situation type would occur in coordinated large-scale networks that reflect situated responding. Whereas networks underlying the social inference and mentalizing involved in responding to a social threat (in regions that make up the “default mode” network) would be reliably more active during social evaluation situations (for reviews of default mode network functions, see Buckner et al., 2008; Barrett and Satpute, 2013)^3^, networks underlying the visuospatial attention and action planning involved in responding to a physical threat would be reliably more active during physical danger situations (for reviews of attention networks, see Chun et al., 2011; Petersen and Posner, 2012; Posner, 2012). These large-scale, distributed networks largely consist of heteromodal regions that engage in the multimodal integration necessary for coordinated interpretation and responding (Sepulcre et al., 2012; Spreng et al., 2013).

As a further test of our second hypothesis, we examined whether participants’ trial-by-trial ratings of immersion during the training session correlated with neural activity, across social evaluation scenarios and across physical danger scenarios. If emotional experience is situated, then feeling immersed in a situation should be realized by neural circuitry that underlies engaging in the specific situation. Whereas immersion in social evaluation situations should occur when affect is grounded in mentalizing about others, immersion in physical danger situations should occur when affect is grounded in taking action in the environment.

## MATERIALS AND METHODS

### PARTICIPANTS

Twenty right-handed, native-English speakers from the Emory community, ranging in age from 20 to 33 (10 female), participated in the experiment. Six additional participants were dropped due to problems with audio equipment (three participants) or excessive head motion in the scanner. Participants had no history of psychiatric illness and were not currently taking any psychotropic medication. They received $100 in compensation, along with anatomical images of their brain.

### MATERIALS

A full and core form of each scenario was constructed, the latter being a subset of the former (see **Table 1**). The full form served to provide a rich, detailed, and affectively compelling scenario. The core form served to minimize presentation time in the scanner, so that the number of necessary trials could be completed in the time available. Each full and core scenario described an emotional situation from a first-person perspective, such that the participant could immerse him- or herself in it. As described shortly, participants practiced enriching the core form of the scenario during the training sessions using details from the full form, so that they would be prepared to immerse in the rich situational detail of the full forms during the scanning session when they received the core forms.

Both situation types were designed so the threat described could be experienced as any number of high arousal, negative emotions like fear or anger (and participants’ ratings of the ease of experiencing negative emotions in the two situation types validated this approach; see Wilson-Mendenhall et al., 2011 for details). In social evaluation situations, another person put the immersed participant in a socially threatening situation that involved damage to his or her social reputation/ego. In physical danger situations, the immersed participant put him- or herself in a physically threatening situation that involved impending or actual bodily harm.

Templates were used to systematically construct different scenarios in each situation type (social evaluation and physical danger). **Table 1** provides examples of the social evaluation and physical danger scenarios. Each template for the full scenarios specified a sequence of six sentences: three primary sentences (P_i_) also used in the related core scenario, and three secondary sentences (S_i_) not used in the core scenario that provided additional relevant detail. The two sentences in each core scenario were created using P_1_ as the first sentence and a conjunction of P_2A_ and P_2C_ as the second sentence.

For the social evaluation scenarios, the template specified the following six sentences in order: P_1_ described a setting and activity performed by the immersed participant in the setting, along with relevant personal attributes; S_1_ provided auditory detail about the setting; P_2A_ described an action (A) of the immersed participant; P_2C_ described the consequence (C) of that action; S_2_ described another person’s action in response to the consequence; S_3_ described the participant’s resulting internal bodily experience. The templates for the physical danger scenarios were similar, except that S_1_ provided visual detail about the setting (instead of auditory), S_2_ described the participant’s action in response to the consequence (instead of another person’s action), and S_3_ described the participant’s resulting external somatosensory experience (on the body surface).

A broad range of real-world situations served as the content of the experimental situations. The physical danger scenarios were drawn from situations that involved vehicles, pedestrians, water, eating, wildlife, fire, power tools, and theft. The social evaluation scenarios were drawn from situations that involved friends, family, neighbors, love, work, classes, public events, and service.

During the training sessions and the critical scan session, 30 social evaluation scenarios and 30 physical danger scenarios were presented. An additional three scenarios of each type were included in the training sessions so participants could practice the scanner task prior to the scan session.

### IMAGING DESIGN

The event-related neuroimaging design involved two critical events: (1) immersing in an emotional scenario (either a social evaluation or physical danger scenario) and (2) experiencing the immersed state in one of four ways upon hearing an auditory categorization cue (as emotional: fearful or angry, or as another active state: planning or observing). We will refer to the first event as “immersion” and the second event as “categorization.” Because all neural patterns described here reflect activity during the first immersion event, we focus on this element of the design (for the categorization results and related methodological details, please see Wilson-Mendenhall et al., 2011). This design afforded a unique opportunity to examine the situations in which emotions emerge before the emotional state was explicitly categorized. As will be described later, the participant could not predict which categorization cue would follow the scenario, so the immersion period reflects situated activity that is not tied to a specific emotion category.

In order to separate neural activity during the immersion events from neural activity during the categorization events, we implemented a catch trial design (Ollinger et al., 2001a, b). Participants received 240 complete trials that each contained a social evaluation scenario or a physical danger scenario followed immediately by one of the four categorization cues. Participants also received 120 partial “catch” trials containing only a scenario (with no subsequent categorization cue), which enabled separation of the first scenario immersion event from the second categorization event. The partial trials constituted 33% of the total trials, a proportion in the recommended range for an effective catch trial design. Each of the 30 social evaluation scenarios and the 30 physical danger scenarios was followed once by each categorization cue, for a total of 240 complete trials (60 scenarios followed by 4 categorizations). Each of the 60 scenarios also occurred twice as a partial trial, for a total of 120 catch trials.

During each of 10 fMRI runs, participants received 24 complete trials and 12 partial trials. The complete and partial trials were intermixed with no-sound baseline periods that ranged from 0 to 12 s in increments of 3 s (average 4.5 s) in a pseudo-random order optimized by optseq2 (Greve, 2002). On a given trial, participants could not predict whether a complete or partial trial was coming, a necessary condition for an effective catch trial design (Ollinger et al., 2001a, b). Participants also could not predict the type of situation or the categorization cue they would hear. Across trials in a run, social evaluation and physical danger situations each occurred 18 times, and each of the 4 categorization cues (anger, fear, observe, plan) occurred 6 times, equally often with social evaluation and physical danger scenarios. A given scenario was never repeated within a run.

### PROCEDURE

The experiment contained two training sessions and an fMRI scan session. The first training session occurred 24–48 h before the second training session, followed immediately by the scan. During the training sessions, participants were encouraged to immerse themselves in all scenarios from a first-person perspective, to imagine the scenario in as much vivid detail as possible, and to construct mental imagery as if the scenario events were actually happening to them. The relation of the full to the core scenarios was also described, and participants were encouraged to reinstate the full scenario whenever they heard a core scenario.

During the first training session, participants listened over computer headphones to the full versions of the 66 scenarios that they would later receive on the practice trials and in the critical scan 24–48 h later, with the social evaluation and physical danger scenarios randomly intermixed. After hearing each full scenario, participants provided three judgments about familiarity and prior experiences, prompted by questions and response scales on the screen. After taking a break, participants listened to the 66 core versions of the scenarios, again over computer headphones and randomly intermixed. While listening to each core scenario, participants were instructed to reinstate the full version that they listened to earlier, immersing themselves fully into the respective scenario as it became enriched and developed from memory. After hearing each core scenario over the headphones, participants rated the vividness of the imagery that they experienced while immersed in the scenario. This task encouraged the participants to develop rich imagery upon hearing the core version. A detailed account of the first training session can be found in Wilson-Mendenhall et al. (2011).

During the second training session directly before the scan, participants first listened to the 66 full scenarios to be used in the practice and critical scans, and rated how much they were able to immerse themselves in each scenario, again hearing the scenarios over computer headphones and in a random order. After listening to each full scenario, the computer script presented the question, “How much did you experience ‘being there’ in the situation?” Participants responded on the computer keyboard, using a 1–7 scale, where one meant not experiencing being there in the situation at all, four meant experiencing being there a moderate amount, and seven meant experiencing being there very much, as if it was actually happening to them. The full scenarios were presented again at this point to ensure that participants were reacquainted with all the details before hearing the core versions later in the scanner. This first phase of the second training session lasted about an hour.

Participants were then instructed on the task that they would perform in the scanner and performed a run of practice trials. During the practice and during the scans, audio events were presented and responses collected using E-prime software (Schneider et al., 2002). On each complete trial, participants were told to immerse in the core version of a scenario as they listened to it, and that they would receive one of four words (anger, fear, observe, plan) afterward. The participant’s task was to judge how easy it was to experience what the word described in the context of the situation. The core scenario was presented auditorily at the onset of a 9 s period, lasting no more than 8 s. The word was then presented auditorily at the onset of a 3 s period, and participants responded as soon as ready. To make their judgments, participants pressed one of three buttons on a button box for not easy, somewhat easy, and very easy. During the practice trials, participants used an E-Prime button box to practice making responses. In the scanner, participants used a Current Designs fiber optic button box designed for high magnetic field environments. Participants were also told that there would be partial trials containing scenarios and no word cues, and that they were not to respond on these trials.

At the beginning of the practice trials, participants heard the same short instruction that they would hear before every run in the scanner: “Please close your eyes. Listen to each scenario and experience being there vividly. If a word follows, rate how easy it was to have that experience in the situation.” Participants performed a practice run equal in length to the runs that they would perform in the scanner. Following the practice run, the experimenter and the participant walked 5 min across campus to the scanner. Once settled safely and comfortably in the scanner, an initial anatomical scan was performed, followed by the 10 critical functional runs, and finally a second anatomical scan. Prior to beginning each functional run, participants heard the same short instruction from the practice run over noise-muffling headphones. Participants took a short break between each of the 8 min 3 s runs. Total time in the scanner was a little over 1.5 h.

### IMAGE ACQUISITION

The neuroimaging data were collected in the Biomedical Imaging Technology Center at Emory University on a research-dedicated 3T Siemens Trio scanner. In each functional run, 163 T2^*^-weighted echo planar image volumes depicting BOLD contrast were collected using a Siemens 12-channel head coil and parallel imaging with an iPAT acceleration factor of 2. Each volume was collected using a scan sequence that had the following parameters: 56 contiguous 2 mm slices in the axial plane, interleaved slice acquisition, TR = 3000 ms, TE = 30 ms, flip angle = 90°, bandwidth = 2442 Hz/Px, FOV = 220 mm, matrix = 64, voxel size = 3.44 mm × 3.44 mm × 2 mm. This scanning sequence was selected after testing a variety of sequences for susceptibility artifacts in orbitofrontal cortex, amygdala, and the temporal poles. We selected this sequence not only because it minimized susceptibility artifacts by using thin slices and parallel imaging, but also because using 3.44 mm in the X–Y dimensions yielded a voxel volume large enough to produce a satisfactory temporal signal-to-noise ratio. In each of the two anatomical runs, 176 T1-weighted volumes were collected using a high resolution MPRAGE scan sequence that had the following parameters: 192 contiguous slices in the sagittal plane, single-shot acquisition, TR = 2300 ms, TE = 4 ms, flip angle = 8°, FOV = 256 mm, matrix = 256, bandwidth = 130 Hz/Px, voxel size = 1 mm × 1 mm × 1 mm.

### IMAGE PREPROCESSING AND ANALYSIS

Image preprocessing and statistical analysis were conducted in AFNI (Cox, 1996). The first anatomical scan was registered to the second, and the average of the two scans computed to create a single high-quality anatomical scan. Initial preprocessing of the functional data included slice time correction and motion correction in which all volumes were registered spatially to a volume within the last functional run. A volume in the last run was selected as the registration base because it was collected closest in time to the second anatomical scan, which facilitated later alignment of the functional and anatomical data. The functional data were then smoothed using an isotropic 6 mm full-width half-maximum Gaussian kernel. Voxels outside the brain were removed from further analysis at this point, as were high-variability low-intensity voxels likely to be shifting in and out of the brain due to minor head motion. Finally, the signal intensities in each volume were divided by the mean signal value for the respective run and multiplied by 100 to produce percent signal change from the run mean. All later analyses were performed on these percent signal change data.

The averaged anatomical scan was corrected for non-uniformity in image intensity, skull-stripped, and then aligned with the functional data. The resulting aligned anatomical dataset was warped to Talairach space using an automated procedure employing the TT_N27 template (also known as the Colin brain, an averaged dataset from one person scanned 27 times).

Regression analyses were performed on each individual’s preprocessed functional data using a canonical, fixed-shape Gamma function to model the hemodynamic response. In the first regression analysis, betas were estimated using the event onsets for 10 conditions: 2 situation immersion conditions (social, physical) and 8 categorization conditions that resulted from crossing the situation with the categorization cue (social-anger, physical-anger, social-fear, physical-fear, social-observe, physical-observe, social-plan, physical-plan). Again, we only present results for the two situation immersion conditions here (see Wilson-Mendenhall et al., 2011 for the categorization results). The two situation immersion conditions were modeled by creating regressors that included scenario immersion events from both the complete trials and the partial trials. Including scenario immersion events from both trial types in one regressor made it possible to mathematically separate the situation immersion conditions from the subsequent categorization conditions (Ollinger et al., 2001a, b). Because scenario immersion events were 9 s in duration, the Gamma function was convolved with a boxcar function for the entire duration to model the situation immersion conditions. Six regressors obtained from volume registration during preprocessing were also included to remove any residual signal changes correlated with movement (translation in the *X*, *Y*, and *Z* planes; rotation around the *X*, *Y*, and *Z* axes). Scanner drift was removed by finding the best-fitting polynomial function correlated with time in the preprocessed time course data.

At the group level, the betas resulting from the each individual’s regression analysis were then entered into a second-level, random-effects ANOVA. Two key analyses were computed at this level of analysis using a voxel-wise threshold of *p* < 0.005 in conjunction with the 41-voxel extent threshold determined by AFNI ClustSim to produce an overall corrected threshold of *p* < 0.05. In the first analysis (that assessed our first hypothesis), we extracted clusters that were more active during immersion in social evaluation situations than in the no-sound baseline and clusters that were more active during immersion in physical danger situations than in the no-sound baseline (using the voxel-wise and extent thresholds specified above). We then entered the results of these two contrasts (social evaluation > baseline; physical danger > baseline) into a conjunction analysis to determine clusters shared by the two situation types (i.e., overlapping regions of activity). In the second analysis (that assessed our second hypothesis), we computed a standard contrast to directly compare immersion during social evaluation situations to immersion during physical danger situations using *t* tests (social evaluation > physical danger; physical danger > social evaluation).

A second individual-level regression was computed to examine the relationship between neural activity and the scenario immersion ratings collected during the training session just prior to the scan session, providing an additional test of our second hypothesis. This regression model paralleled the first regression model with the following exceptions. In this regression analysis, each participant’s “being there” ratings were specified trial-by-trial for each scenario in the social evaluation immersion condition and in the physical danger immersion condition. For the two situation immersion conditions (social evaluation and physical danger), both the onset times and ratings were then entered into the regression using the amplitude modulation option in AFNI. This option specified two regressors for each situation immersion condition, which were used to detect: (1) voxels in which activity was correlated with the ratings (also known as a parametric regressor); (2) voxels in which activity was constant for the condition and was not correlated with the ratings.

At the group level, each participant’s betas produced from the first parametric regressor for each situation immersion condition (i.e., indicating the strength of the correlation between neural activity and “being there” immersion ratings) were next entered into a second-level analysis. In this analysis, the critical statistic for each condition was a *t* test indicating if the mean across individuals differed significantly from zero (zero indicating no correlation between neural activity and the ratings). In these analyses, a slightly smaller cluster size of 15 contiguous voxels was used in conjunction with the voxel-wise threshold of *p* < 0.005.

In summary, this analysis is examining whether scenarios rated as easier to immerse in during the training are associated with greater neural activity in any region of the brain (the individual-level analysis), and whether this relationship between immersion ratings and neural activity is consistent across participants (group-level analysis). We computed this analysis separately for social evaluation and for physical danger situation types to test our hypothesis. This analysis is not examining between-subject individual differences in immersion (i.e., whether participants who generally experience greater immersion across all scenarios also show greater neural activity in specific regions), which is a different question that is not of interest here.

## RESULTS

### COMMON NEURAL ACTIVITY DURING IMMERSION ACROSS SITUATIONS

**FIGURE 2:**
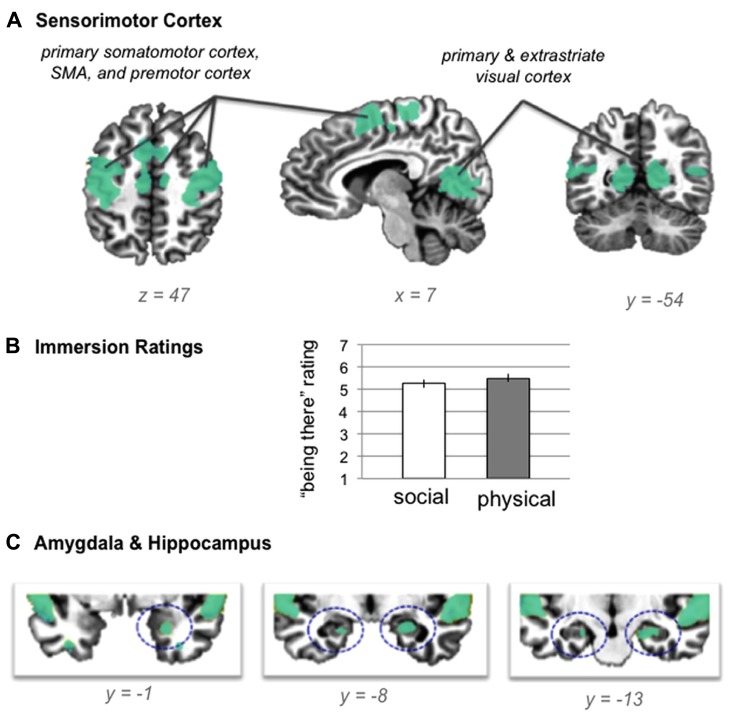
**(A)** shared neural activity during social evaluation and physical danger situations in sensorimotor cortex (revealed by the conjunction analysis in which each situation was compared to the “no sound” baseline) **(B)** self- reported immersion ratings from the training session (error bars depict SEM across participant condition means) **(C)** shared neural activity revealed by the conjunction analysis in the amygdala and hippocampus.

Our first hypothesis was that neural activity during both situations would be reliably greater than baseline across multiple sensory modalities and across regions involved in detecting and integrating salient sensory information (see **Table 2** for the baseline contrasts). As shown in **Figure 2A**, neural activity was reliably greater than baseline in bilateral primary somatomotor and visual cortex, as well as premotor cortex, SMA, and extrastriate visual cortex, suggesting that participants easily immersed in the situations. The self-reported rating data from the training session confirmed that participants found the social evaluation and physical danger situations relatively easy to immerse in (see **Figure 2B**), with no significant differences in “being there” ratings between situation types [repeated measures *t* test; *t*(19) = 1.64, *p* > 0.05]. Because participants listened to the scenarios with their eyes closed and because participants did not make responses while immersing in the scenarios, it is significant that these sensorimotor regions were significantly more active than the no-sound baseline. As would be expected with an auditory, language-based immersion procedure, we observed activity in bilateral auditory cortex and in superior temporal and inferior frontal regions associated with language processing, with more extensive activity in the left frontal regions.

**Table 2:** Social evaluation > baseline and physical danger > baseline contrasts.

**Cluster**	**Brain region**	**Brodmann area(s)**	**Mean *t***	**Spatial extent**	**Peak**		
					***x***	***y***	***z***
**Social evaluation > baseline**							
1	R temp pole/STG/STS	38, 21, 22, 41, 42	4.73	1868	46	-16	-7
	R angular g	39					
	R ITG/fusiform g	37, 19					
	R mid/sup occipital g	19					
2	L temp pole/STG/STS	38, 21, 22, 41, 42	4.83	1780	-45	9	-15
	L angular g	39					
	L mid/sup occipital g	19					
3	L and R calarine/lingual g	17, 18, 19	4.21	1532	14	-57	14
	L and R posterior cingulate	31					
	L and R parahippocampal g	35, 36					
	L and R hippocampus/amygdala						
4	L premotor/precentral g	6, 4	4.36	921	-37	-6	50
	L postcentral g	2, 3					
	L lateral PFC/Ant insula	44, 45, 46, 9					
5	L and R SMA/precentral g	6, 4	4.60	596	-4	7	48
6	R premotor/precentral g	6, 4	4.52	501	50	-11	51
	R postcentral g	2, 3					
7	mPFC/mOFC	10, 11	4.45	115	-1	34	-8
8	R lateral PFC	45/46	4.09	77	52	19	22
9	L fusiform g	37	4.00	58	-36	-38	-11
**Phyiscal danger > baseline**							
1	L and R SMA/premotor	6	4.37	6887	-5	7	47
	L and R precentral g	4					
	L and R postcentral g	2, 3					
	L and R mid cingulate	24, 31					
	L lateral PFC/Ant insula	44, 45, 46, 9					
	L and R temp Pole/STG/STS	38, 21, 22, 41, 42					
	L and R MTG	37					
	L ITG/fusiform	37					
	L and R parahippocampal g	35, 36					
	L and R hippocampus/amygdala						
	L and R mid/sup occipital g	19					
	L and R calcarine/lingual g	17, 18, 19					
	L inferior parietal	40, 7					
2	L and R thalamus		4.22	85	-9	-21	2
3	R lateral PFC	45/46	3.93	68	55	19	26

*Spatial extent is the number of 23.67 mm^3^ functional voxels. L is left and R is right, Ant is anterior, Mid is middle, Sup is superior, m is medial, and g is gyrus. PFC is prefrontal cortex and OFC is orbitofrontal cortex. STG is superior temporal gyrus, STS superior temporal sulcus, MTG is middle temporal gyrus, and ITG is inferior temporal gyrus. SMA is supplementary motor area.*

Consistent with the hypothesis that immersion would also generally involve selection, encoding, and integration of salient sensory and other information, we observed activity in bilateral hippocampus and in right amygdala (see **Figure 2C**). Extensive evidence implicates the hippocampus in mnemonic functions (Squire and Zola-Morgan, 1991; Tulving, 2002; Squire, 2004), especially the integration and binding of the multimodal information involved in constructing (and reconstructing) situated memories (Addis and McAndrews, 2006; Kroes and Fernandez, 2012). More recent evidence establishes a central role for this structure in simulating future, imagined situations (Addis et al., 2007; Hassabis et al., 2007; Schacter et al., 2007, 2012), which is similar in nature to our immersion paradigm, and which requires similar integration and binding of concepts established in memory (from prior experience). The amygdala plays a central role in emotional experiences by efficiently integrating multisensory information to direct attention and guide encoding (Costafreda et al., 2008; Bliss-Moreau et al., 2011; Klasen et al., 2012; Lindquist et al., 2012), especially during situations that involve threat (Adolphs, 2008; Miskovic and Schmidt, 2012). As we will see, no differences emerged in the amygdala or in the hippocampus during the social evaluation and physical danger situations, suggesting these structures played a similar role in both types of experiences.

### UNIQUE NEURAL PATTERNS EMERGE FOR SOCIAL EVALUATION AND PHYSICAL DANGER SITUATIONS

**FIGURE 3:**
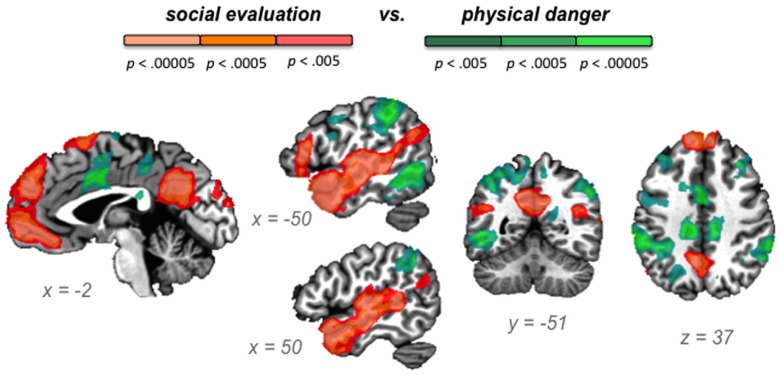
**Social evaluation vs. physical danger contrast, with regions reliably more active during social evaluation in orange and regions reliably more active during physical danger in green**.

**FIGURE 4:**
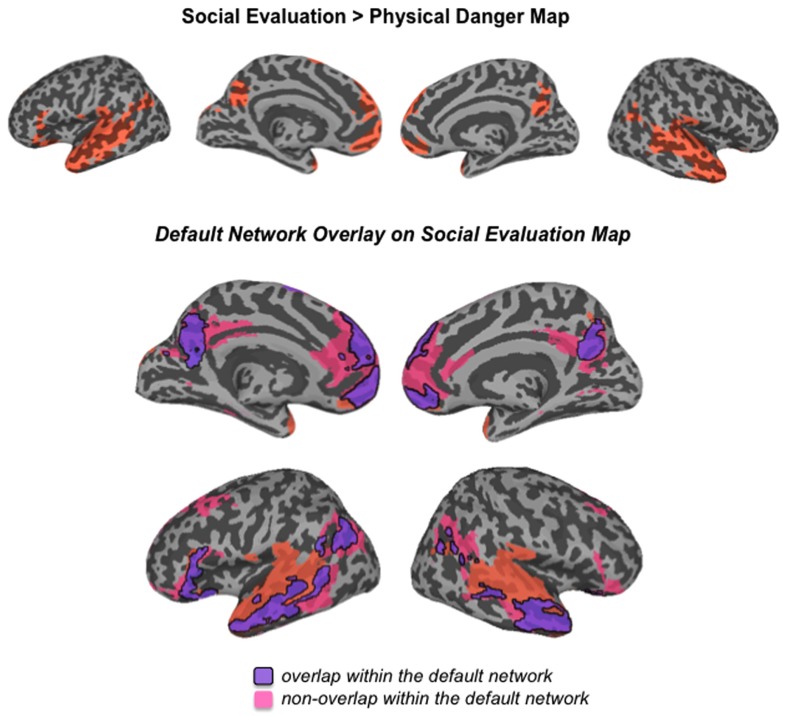
**Comparison of the social evaluation map from this study with the default mode network defined by Yeo et al. (2011)**.

**FIGURE 5:**
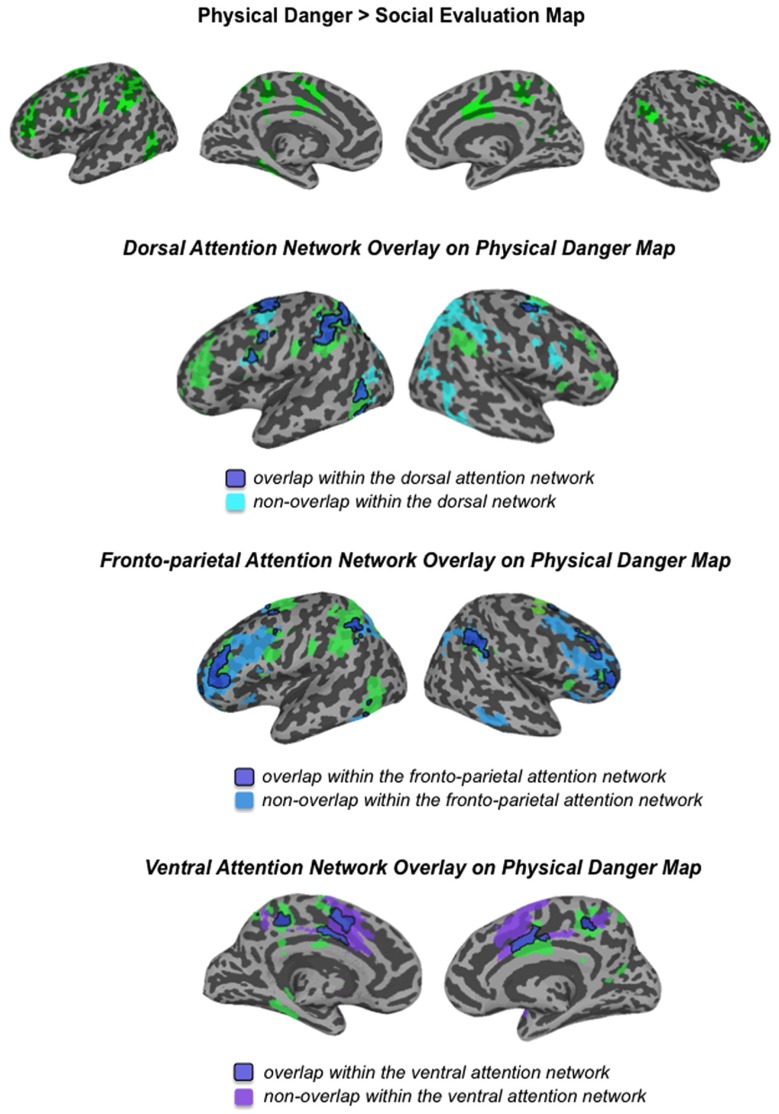
**Comparison of the physical danger map from this study with the attention networks defined by Yeo et al. (2011)**.

Our second hypothesis was that networks underlying the social inference and mentalizing involved in responding to a social threat would be reliably more active during social evaluation situations, whereas networks underlying visuospatial attention and action planning involved in responding to a physical threat would be reliably more active during physical danger situations. As **Table 3**, together with **Figures 3–5**, illustrate, the neural patterns that emerged when we compared social evaluation situations to physical danger situations are consistent with these predictions. **Figure 3** shows these results on representative 2D slices, with regions showing reliably greater activity during social evaluation in orange, and regions showing reliably greater activity during physical danger in green. **Figures 4 and 5** display these maps projected onto the surface of the brain^4^, and directly compare the maps from this study with the large-scale networks that have been defined using resting state connectivity techniques across large samples (Yeo et al., 2011).

#### Heightened activity in the default mode network during social evaluation

As displayed in **Figure 3** and **Table 3**, robust activity was observed during immersion in social evaluation situations (vs. physical danger situations) in midline medial prefrontal and posterior cingulate regions, as well as lateral temporal regions, in which activity spanned from the temporal pole to the posterior superior temporal sulcus/temporoparietal junction bilaterally, and on the left, extended in to inferior frontal gyrus. This pattern of activity maps onto a network that is often referred to as the “default mode” network (Gusnard and Raichle, 2001; Raichle et al., 2001; Buckner et al., 2008). **Figure 4** illustrates the overlap between the default mode network and the pattern of neural activity that underlies immersing in social evaluation situations here (Yeo et al., 2011). The default mode network has been implicated in mentalizing and social inference (i.e., inferring what others’ are thinking/feeling and how they will act), as well as other socially motivated tasks, including autobiographical memory retrieval, envisioning the future, and moral reasoning (for reviews, see Buckner et al., 2008; Van Overwalle and Baetens, 2009; Barrett and Satpute, 2013). Consistent with the idea of situated emotional experience, participants engaged in the social inference and mentalizing that would be adaptive in responding to a social threat when immersed in social evaluation situations.

**Table 3:** Brain regions that emerged in the social evaluation vs. physical danger contrast.

Cluster	Brain region	Brodmann area(s)	Mean *t*	Spatial extent	Peak		
					*x*	*y*	*z*
**Social evaluation > physical danger**							
1	L STG/STS/post insula/angular g/temp pole/OFC/IFG	41, 42, 22, 21, 39, 38, 47, 45	5.13	2059	-58	-17	-1
2	R STG/STS/post insula/temp pole	41, 42, 22, 21, 38******	4.77	1668	51	9	-20
3	mPFC/mOFC/SMA	10, 11, 9, 8, 6	4.63	1136	4	51	31
4	Post cingulate/precuneus	31, 7	4.73	498	-7	-53	34
5	R STG/STS/angular g	22, 39	3.97	112	40	-49	22
6	L cuneus	18	3.67	57	-7	-95	23
**Physical danger > social evaluation**							
1	L inf/sup parietal/precuneus	40, 7	4.23	992	-59	-33	38
2	Mid cing/L premotor/L MFG	24, 6	4.20	715	4	6	31
3	L MTG/fusiform g/parahippocampal g	37, 20, 35	4.37	478	-49	-54	0
4	Mid cing	31, 23	4.35	321	-13	-26	37
5	L MFG	46, 9, 10	4.14	266	-37	38	16
6	R MFG/Ant insula/OFC	10	3.99	212	37	44	6
7	R inf parietal	40	4.14	199	59	-37	35
8	R premotor	6	4.16	173	15	2	59
9	R MFG	9	3.95	104	31	30	38
10	R precuneus	7	3.94	74	7	-56	53
11	L OFC	11	3.82	49	-29	44	-5
12	R restrosplenial	29	3.78	42	12	-44	12

*Spatial extent is the number of 23.67 mm^3^ functional voxels. L is left and R is right. Post is posterior, Ant is anterior, Inf is inferior, Sup is superior, m is medial, and g is gyrus. PFC is prefrontal cortex, OFC is orbitofrontal cortex, Cing is cingulate, and MFG is middle frontal gyrus. STG is superior temporal gyrus, STS is superior temporal sulcus, and MTG is middle temporal gyrus. SMA is supplementary motor area.*

#### Heightened activity in fronto-parietal attention networks during physical danger

**Figure 3** and **Table 3** show the fronto-parietal patterns of activity observed during immersion in physical danger situations (vs. social evaluation situations). In addition to lateral frontal and parietal regions (including bilateral middle frontal gyrus, bilateral inferior frontal gyrus extending into pars orbitalis, bilateral inferior parietal lobule, and bilateral superior parietal/precuneus), neural activity was also reliably greater in right anterior insula, mid cingulate cortex, and bilateral premotor cortex during immersion in physical danger situations. **Figure 5** illustrates the overlap between this pattern of activity and three networks that have been implicated in attention^5^ (Chun et al., 2011; Petersen and Posner, 2012; Posner, 2012). The most significant overlap was observed in the lateral fronto-parietal executive network and the dorsal attention network. These networks are thought to allocate attentional resources to prioritize specific sensory inputs (what is often referred to as “orienting” to the external environment) and to guide flexible shifts in behavior (Dosenbach et al., 2007; Petersen and Posner, 2012). The operations they carry out are critical for maintaining a vigilant state (Tang et al., 2012), which is important during threat. Less overlap was evident in the ventral attention network that is thought to interrupt top-down operations through bottom-up “salience” detection (Corbetta et al., 2008), although robust activity was observed in the mid cingulate regions shown in **Figure 5** that support the action monitoring that occurs, especially, in situations involving physical pain (Morecraft and Van Hoesen, 1992; Vogt, 2005). Taken together, this pattern of results suggests, strikingly, that immersion in the physical danger situations (from a first-person perspective with eyes closed) engaged attention networks that are studied almost exclusively using external visual cues. Consistent with the idea of situated emotional experience, participants engaged in the monitoring of the environment and preparation for flexible action that would be adaptive in action to a physical threat when immersed in physical danger situations.

#### Immersion ratings correlate with activity in different regions during social evaluation vs. physical danger situations

**FIGURE 6:**
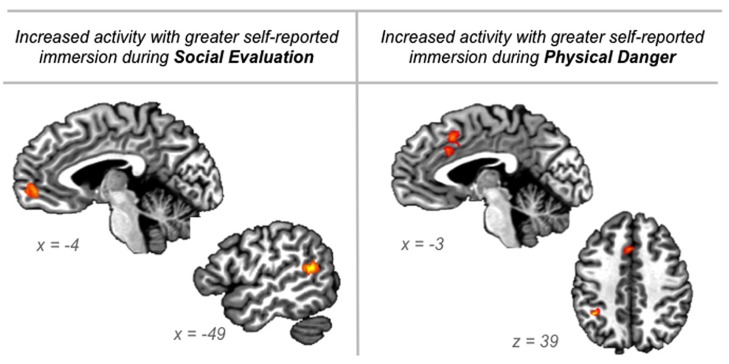
**Regions in which neural activity was significantly correlated with participants’ “being there” ratings of immersion collected during the training session just prior to scanning, for the social evaluation situations and for the physical danger situations**.

To provide another test of our second hypothesis, we examined whether self-reported immersion ratings of “being there” in the situation (from the training session) were associated with brain activity during the two situation types. If emotional experience is situated, then feeling immersed in a situation should be realized by neural circuitry that underlies engaging in the specific situation. Whereas immersion in social evaluation situations should occur when affect is grounded in mentalizing about others, immersion in physical danger situations should occur when affect is grounded in taking action in the environment. The results displayed in **Figure 6** support this prediction.

During social evaluation situations, participants’ immersion ratings correlated with activity in anterior medial prefrontal cortex (frontal pole area; peak voxel -6 51 0; 23 voxels) and in superior temporal gyrus/sulcus (peak voxel -47 -49 14; 24 voxels; see **Figure 6**). As described above, these regions are part of the default mode network and are central to social perception and mentalizing (Allison et al., 2000; Buckner et al., 2008; Adolphs, 2009; Van Overwalle, 2009). The anterior, frontal pole region of medial prefrontal cortex is considered the anterior hub of the default mode network (Andrews-Hanna et al., 2010) that integrates affective information from the body with social event knowledge (including inferences about others’ thoughts) originating in ventral and dorsal aspects of medial prefrontal cortex, respectively (Mitchell et al., 2005; Krueger et al., 2009). This integration may underlie the experience of “personal significance” (Andrews-Hanna et al., 2010) that appears important for immersing in social evaluation situations.

In contrast, during physical danger situations, participants’ immersion ratings correlated with activity in dorsal anterior cingulate/mid cingulate (extending into SMA; peak -1 17 40; 40 voxels) and in left inferior parietal cortex (peak -36 -46 39; 15 voxels; see **Figure 6**). The robust cluster of activity that emerged in the cingulate is part of the ventral attention “salience” network, and it is anterior to the mid cingulate activity observed in the initial whole-brain contrasts reported above. Because this region has been implicated across studies of emotion, pain, and cognitive control, and because it is anatomically positioned at the intersection of insular-limbic and fronto-parietal sub-networks within the attention system, it may play an especially important role in specifying goal-directed action based on affective signals originating in the body (Shackman et al., 2011; Touroutoglou et al., 2012). This integration may underlie the experience of action-oriented agency (Craig, 2009) that appears important for immersing in physical danger situations. The significant correlation with activity in left inferior parietal cortex, which supports planning action in egocentric space (e.g., Fogassi and Luppino, 2005), further suggests that immersion in physical danger situations is driven by preparing to act in the environment.

## DISCUSSION

Our novel scenario immersion paradigm revealed robust patterns of neural activity when participants immersed themselves in social evaluation scenarios and in physical danger scenarios. Consistent with participants’ high self-reported immersion ratings, neural activity across multiple sensory regions, and across limbic regions involved in the multisensory integration underlying the selection, encoding, and interpretation that influences what is salient and remembered (e.g., amygdala, hippocampus), occurred during both situation types. In addition to this shared activity, distributed patterns unique to each situation type reflected situated responding, with regions involved in mentalizing and social cognition more active during social evaluation and with regions involved in attention and action planning more active during physical danger.

Taken together, these findings suggest that our method produced vivid, engaging experiences during neuroimaging scans and that it could be used to study a variety of emotional experiences. One reason this immersion paradigm may be so powerful is that people often find themselves immersed in imagined situations in day-to-day life. Large-scale experience sampling studies have revealed that people spend much of their time imagining experiences that are unrelated to the external world around them (e.g., Killingsworth and Gilbert, 2010). An important direction for future research will be to understand if, consistent with other imagery-based paradigms, physiological changes occur during our scenario immersion paradigm and if these physiological changes are associated with subjective experiences of immersion.

The scenarios we developed for this study represent a small subset of the situations that people experience in real life (see also Wilson-Mendenhall et al., 2013). Because emotional experiences vary tremendously, it is adaptive to develop situated knowledge that guides inference and responding when similar situations arise in the future (Barsalou, 2003, 2008, 2009; Barrett, 2013). Here, we focused on immersion in emotion-inducing situations before they were explicitly categorized as an emotion (or another state). From our perspective, the situation plays a critical role in the emergence of an emotion, and it should not be considered a separate phenomenon from it (Barrett, 2009b, 2012; Wilson-Mendenhall et al., 2011). For example, it would be impossible to experience *fear* upon delivering a public speech without inferring others’ thoughts. Instead of viewing mentalizing as a “cold” cognitive process that interacts with a primitive “hot” emotion, we view mentalizing as an essential part of the situation in which the emotion emerges. Likewise, it would be impossible to experience *fear* upon getting lost in the woods without focusing attention on the environment (in other words, if one was instead lost in internal thought while traversing the same environment, it is unlikely that this fear would occur). We propose that it will be more productive to study emotional experience as dynamic situated conceptualizations that the brain continually generates to interpret one’s current state (based on prior experience), as opposed to temporally constrained cognition-emotion frameworks that often strip away much of the dynamically changing situated context. A situated approach also offers new insights into studying dynamic emotion regulation and dysregulation (Barrett et al., in press).

Network approaches to brain function provide functional frameworks for interpreting the distributed patterns that characterize situated experiences (Cabral et al., 2011; Deco et al., 2011; Lindquist and Barrett, 2012; Barrett and Satpute, 2013). As shown in **Figures 4 and 5**, the patterns unique to each situation type in this study can be differentiated by the anatomically constrained resting state networks^6^ identified in previous work (Raichle et al., 2001; Fox et al., 2005; Vincent et al., 2006; Dosenbach et al., 2007; Fair et al., 2007; Seeley et al., 2007; Yeo et al., 2011; Touroutoglou et al., 2012). Whereas the neural patterns underlying social threat situations primarily map onto the default mode network that supports social inference and mentalizing, the neural patterns underlying physical threat situations primarily map onto attention networks underlying monitoring of the environment and action planning. The neural pattern unique to each situation type reflects adaptive, situated responding. Furthermore, regions traditionally associated with emotion diverged in line with these networks (e.g., ventromedial prefrontal cortex as part of the default mode network; lateral orbitofrontal cortex and cingulate regions as part of the attention networks). Interestingly, these regions appear to be central to immersion in each type of situation, with the anterior medial prefrontal cortex (which is often considered part of ventromedial prefrontal cortex) associated with immersion during social evaluation situations and dorsal anterior cingulate associated with immersion during physical danger situations. These results suggest, strikingly, that the brain realizes immersion differently depending on the situation.

Resting state networks provide a starting point for examining how networks underlie situated experiences, but recent evidence suggests that coordination between regions in these networks dynamically changes during different psychological states (e.g., van Marle et al., 2010; Raz et al., 2012; Wang et al., 2012). In this study, for example, the neural patterns underlying physical danger experiences recruited various aspects of several different attention networks. Attention is primarily studied using simple visual detection tasks that examine external stimuli vs. internal goal dichotomies. Recent reviews emphasize the need for research that examines how attention systems operate during experiences guided by memory (e.g., Hutchinson and Turk-Browne, 2012), which arguably constitute much of our experience. Because inferior parietal cortex and cingulate regions figured prominently in the pattern observed across the attention networks in this study, this particular configuration may reflect the attention operations involved in coordinating bodily actions in space. It is also important to consider that these patterns reflect relative differences between the social and physical threat situations. As we showed initially, the situation types also share patterns of activity that contribute to the overall pattern of situated activity. In our view, it is useful to think about situated neural activity as dynamically changing patterns that are distributed across structurally and functionally distinct networks (see also Barrett and Satpute, 2013). Even within a structurally defined network, different distributed patterns of neural activity may reflect unique functional motifs that underlie different experiences and behaviors (Sporns and Kotter, 2004).

In closing, a psychological construction approach to studying situated emotion motivates different questions than traditional approaches to studying emotion. It invites shifting research agendas from defining five or so emotion categories to studying the rich situations that characterize emotional experiences.

## Conflict of Interest Statement

The authors declare that the research was conducted in the absence of any commercial or financial relationships that could be construed as a potential conflict of interest.

## Footnotes

1 This meta-analytic database has recently been updated to include articles through 2011. The proportions reported here reflect the updated database.

2 Lindquist etal. (2012) distinguished between “emotion perception” (defined as perception of emotion in others) and “emotion experience” (defined as experience of emotion in oneself) in their meta-analysis. When restricting our analysis of study methods to studies that involved emotion experience (as coded in the database), the use of imagery methods was still minimal (10% of 233 studies). Although emotional imagery is typically thought of as an induction of emotion experience, it seems likely that imagined situations, especially if they are social in nature, involve dynamic emotion perception as well.

3 There is substantial evidence that default mode network (DMN) regions are active during tasks that involve social inference and mentalizing (for reviews, see Barrett and Satpute, 2013; Buckner and Carroll, 2007; Van Overwalle and Baetens, 2009) and that the DMN is disrupted in disorders involving social deficits (for reviews, see Menon, 2011; Whitfield-Gabrieli and Ford, 2012). Recent work has directly demonstrated that neural activity during social/mentalizing tasks occurs in the DMN as it is defined using resting state analyses (e.g., Andrews-Hanna etal., 2010) and that resting state connectivity in the DMN predicts individual differences in social processing (e.g., Yang etal., 2012).

4 It is important to note that each individual’s data were not analyzed on the surface. We are using a standardized (Talairach) surface space for illustration of the group results in comparison to the resting state network maps from a large sample that have been made freely available (Yeo etal., 2011).

5 These networks are sometimes referred to by different names, and can take somewhat different forms depending on the methods used to define them (with core nodes remaining the same). Because the network maps we present here are taken from Yeo etal. (2011), we use their terminology. They note (and thus so do we) that the ventral attention network, especially, is similar to what has been described as the salience network (Seeley etal., 2007) and the cingulo-opercular network (Dosenbach etal., 2007).

6 The term “resting state” is often misinterpreted to mean the resting brain. It should not be assumed that the brain is actually “at rest” during these scans, but simply that there is no externally orienting task.
